# Impact of the pneumococcal 10-valent vaccine on reducing hospitalization for community-acquired pneumonia in children

**DOI:** 10.1016/j.rppede.2016.03.008

**Published:** 2016

**Authors:** Sandra Rodrigues da Silva, Luane Marques de Mello, Anderson Soares da Silva, Altacílio Aparecido Nunes

**Affiliations:** a Superintendência Regional de Saúde de Alfenas, Alfenas, MG, Brazil; b Faculdade de Medicina de Ribeirão Preto, Universidade de São Paulo (FMRP-USP), São Paulo, SP, Brazil

**Keywords:** Pneumonia, Streptococcus pneumoniae, Immunization, Pneumococcal vaccine, Hospitalization, Child

## Abstract

**Objective:**

To describe and analyze the occurrence of hospitalizations for community-acquired pneumonia in children before and after the pneumococcal 10-valent conjugate vaccine implementation into the National Immunization Program.

**Methods:**

This is an ecological study that includes records of children younger than one year old, vaccinated and not vaccinated with the pneumococcal 10-valent conjugate vaccine in the periods pre- and post-inclusion of the vaccine in the National Immunization Program in the area covered by the Regional Health Superintendence of Alfenas, state of Minas Gerais, Brazil. Vaccination was considered as the exposure factor and hospitalization for community-acquired pneumonia as the endpoint, using secondary annual data by municipality. The prevalence ratio and its 95% confidence interval (95%CI) were used to verify the association between variables. The Z test was used to calculate the difference between proportions.

**Results:**

Considering the 26 municipalities of the Regional Health Superintendence of Alfenas, there was a significant reduction in hospitalizations for community-acquired pneumonia in children younger than one year of age, with prevalence ratio (PR)=0.81 (95%CI: 0.74-0.89; *p*<0.05), indicating a 19% lower prevalence of hospitalization for community-acquired pneumonia in the post-vaccination period.

**Conclusions:**

The results suggest the effectiveness of the pneumococcal 10-valent conjugate vaccine in preventing severe cases of community-acquired pneumonia in children younger than one year of age.

## Resumo

**Objetivo:**

Descrever e analisar a ocorrência de internações por pneumonia adquirida na comunidade em crianças antes e após a implantação, no Programa Nacional de Imunização, da vacina pneumocócica 10-valente (conjugada).

**Métodos:**

Trata-se de um estudo ecológico que incluiu registros de crianças menores de um ano, vacinadas e não vacinadas com a vacina antipneumocócica 10-valente conjugada, no período pré e pós-inclusão da vacina no Programa Nacional de Imunização na área de abrangência da Superintendência Regional de Saúde de Alfenas, MG, Brasil. A vacinação foi considerada como fator de exposição e a hospitalização por pneumonia adquirida na comunidade como desfecho, com o uso de dados anuais secundários por município. Para verificar a associação entre as variáveis foi empregada a razão de prevalência e seu intervalo de confiança 95% (IC95%). Para o cálculo de diferença entre proporções empregou-se o teste Z.

**Resultados:**

Considerando os 26 municípios da Superintendência Regional de Saúde de Alfenas, houve redução significativa do número de hospitalização por pneumonia adquirida na comunidade em crianças abaixo de um ano, com razão de prevalência (RP)=0,81 (IC95% 0,74-0,89; *p*<0,05), o que indica uma prevalência de internação por pneumonia adquirida na comunidade 19% menor no período pós-vacinal.

**Conclusões:**

Os resultados sugerem a efetividade da vacina pneumocócica 10-valente (conjugada) na prevenção de casos graves da pneumonia adquirida na comunidade em crianças menores de um ano.

## Introduction

Among the various bacterial etiologic agents involved in the genesis of community-acquired pneumonia (CAP), *Streptococcus pneumoniae* is the main cause of the disease in children and adults. Currently, there are reports of 91 serotypes^1^^,^ 2 associated with CAP and other diseases. However, CAP causes greater morbidity and its occurrence is estimated at 13.8 million new cases worldwide each year.^3^^,^ 4

Between 6% and 16% of CAP cases require hospitalization, and in children under age five, the disease, particularly of bacterial etiology, accounts for 20-40% of admissions only in the Americas.^4^ In Brazil, between 2004 and 2006, pneumococcal diseases were responsible for about 34,000 hospitalizations.

The global estimate of CAP incidence among children aged 1-5 years in developing countries is 0.29 episodes per child-year. This is equivalent to an annual incidence of about 150 million cases; 11-20% of these cases require hospitalization.^4^ In Brazil, according to the Hospital Information System (SIH-SUS), from January 2005 to March 2006, CAP accounted for about 20% of the hospitalizations of children under age five.^5^^-^ 7

Consensually, preventive measures are the best way to reduce the incidence of pneumococcal disease and its consequences, such as hospitalization and premature death.^5^ Among these measures, active immunization against the main causative agents, particularly certain serotypes of *Streptococcus pneumoniae*, has proven highly efficient over the years in reducing the occurrence of severe disease.^5^^,^ 6

In Brazil, with the incorporation of pneumococcal vaccines, particularly 10-valent, since 2010,^6^^,^ 7 there is a reduction of pneumococcal diseases, such as meningitis and CAP,^8^^-^ 10 in children under two years of age, with a decline in the number of hospitalizations, malnutrition, deaths, and parental work absenteeism, as well as cost reduction, among others.^11^^,^ 12

The aim of the present paper was to evaluate the impact of 10-valent pneumococcal conjugate vaccine (PCV-10) before booster shot in hospitalized children under one year of age with CAP, before and after its introduction in the National Immunization Program (PNI) in municipalities belonging to the Regional Health Superintendence of Alfenas (SRS/Alfenas, MG, Brazil).

## Method

This is an ecological study including records of children under one year old who received or not 10-valent pneumococcal conjugate vaccine in the period before and after its inclusion in the PNI, in which vaccination was considered as the exposure factor and CAP hospitalization as the endpoint. The study used secondary annual data by municipality to calculate vaccination coverage and pneumonia morbidity rates in children under one year old from 2007 to 2013. Comparison of raw numbers (i.e., non-proportional numbers) of CAP hospitalization from 2007 to 2013 in the municipalities belonging to SRS/Alfenas was performed.

We assessed records of children up to one year old, living in 26 municipalities under the jurisdiction of SRS/Alfenas—State Department of Health of Minas Gerais, Brazil. Data were collected through search in the Information System of the National Immunization Program (SI-PNI)^13^ and Tabwin database available in the Regulation Center of SRS/Alfenas, MG. The municipalities belonging to SRS/Alfenas are: Alfenas, Alterosa, Arceburgo, Areado, Bandeira do Sul, Botelhos, Cabo Verde, Campestre, Campo do Meio, Campos Gerais, Carmo do Rio Claro, Carvalhópolis, Conceição da Aparecida, Divisa Nova, Fama, Guaranésia, Guaxupé, Juruaia, Machado, Monte Belo, Muzambinho, Nova Resende, Paraguaçu, Poço Fundo, São Pedro da União, and Serrania.

During the period mentioned above, the entire child population was included. To verify the association between variables (independent and response), univariate analyzes were used, with measurement of the relative prevalence (RP) ratio and its respective confidence interval of 95% (95%CI) as association estimators. Subsequently, a multivariate logistic regression analysis was performed and all explanatory variables were included in the model (number of pediatric CAP hospitalizations, adjusted age, sex, municipality of residence, vaccination status, vaccination coverage). Chi-square test was used for difference between proportions and independent samples *t* test for difference between means. For all analyzes, a 5% significance level was considered. For storage and analysis of data, the statistical software SPSS 20.0^®^ (IBM SPSS Statistics for Windows, Version 20.0, Armonk, NY, USA) was used.

The research project was submitted to the Institutional Review Board of the Hospital das Clínicas da Faculdade de Medicina de RibeirãoPreto, USP, and was approved on March 19, 2013 (CAAE No: 12487113.5.0000.5440).

## Results

This study aimed to evaluate the number of CAP hospitalization among children under one year old, so before the booster shot, which should take place between 12 and 15 months, and vaccination coverage, which occurred in the period that comprised the pre- (2007, 2008 and 2009) and post- (2011, 2012 and 2013) introduction of PCV-10 in the immunization schedule of PNI recommended by the Ministry of Health. Table 1 shows the number of children in each of the years in the pre- and post-vaccination periods, as well as the number of children included in the study. One should note that, in the period prior to the introduction of PCV-10, the losses were 11.3%, while in the post-vaccine period they were 8.3%. All losses were due to inconsistent records.

**Table 1:** Distribution of children under one year old enrolled in the 26 municipalities of the SRS/Alfenas, MG; in pre- and post-vaccination periods, as well as raw and mean numbers of children included in the study with their respective losses by period.

Periods						
Pre-vaccination			PCV-10 Introduction	Post-vaccination		
2007	2008	2009	2010	2011	2012	2013
5969	5622	5628	5765	5669	5723	5667
	Mean (A)		-		Mean (B)	
	5740		5765		5686	
Records (PNI) - SRS/Alfenas, MG						
2007	2008	2009	2010	2011	2012	2013
5107	5088	5080	5765	5215	5212	5213
	Mean (C)		-		Mean (D)	
	5091		-		5213	
	Losses A-C (%)				Losses B-D (%)	
	11.3		-		8.3	

The gross number of the male and female children records between 2007 and 2009 was, respectively, 2632 and 2459 (*p*<0.05), with an average of nine months (±1) for females and 10 months (±1) for males (*p*<0.05). Considering the period of 2011-2013, there were 2737 records of male and 2476 records of female children (*p*<0.05), with respective averages of 9.5±1 months and 10±1.2 months. There was a significant difference (*p*<0.05).

**Figure 1:**
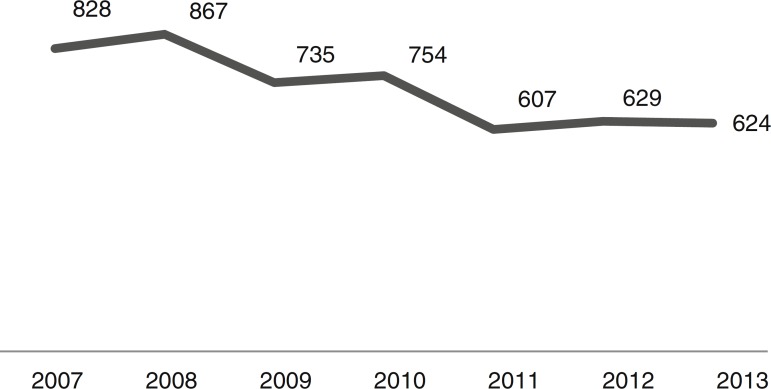
Number of hospitalizations for community-acquired pneumonia (CAP) in children under one year old in 26 municipalities from 2007 to 2013. Coordenadoria de Regulação da SRS/Alfenas, MG, Brazil.

The number of admissions during the study period is shown in Fig. 1. As it can be noted, the number of CAP admissions in the age group under one year old went from 828 in 2007 to 624 in 2013. There were 5044 cases of CAP admissions during the analysis period.

In March 2010, there was the 10-valent pneumococcal conjugate vaccine introduction in the vaccination schedule of children in PNI. Thus, the vaccination coverage that year did not reach the 95% expected coverage (Table 2). Therefore, vaccination coverage in 2010 was not evaluated because it could bias the analysis, both due to reduced coverage and the possibility of delay in vaccine seroconversion.

**Table 2:** Distribution of 10-valent pneumococcal conjugate vaccine coverage in children in 2010, 2011, 2012, and 2013 - SRS/Alfenas, MG, Brazil.

Municipality	2010			2011			2012			2013	
	n	(%)		n	(%)		n	(%)		n	(%)
Alfenas	524	54.24		937	100.64		843	90.55		927	99.57
Alterosa	85	49.13		150	88.24		184	108.24		165	97.06
Arceburgo	23	22.55		76	73.08		117	112.50		99	95.19
Areado	94	45.41		171	97.16		169	96.02		199	113.07
Bandeira do Sul	12	19.35		46	75.41		80	131.15		92	150.82
Botelhos	53	34.42		185	156.78		164	138.98		177	150.00
Cabo Verde	86	49.14		199	125.16		140	88.05		141	88.68
Campestre	115	49.36		215	101.90		184	87.20		235	111.37
C. do Meio	52	45.22		142	112.70		161	127.78		121	96.03
C. Gerais	183	55.62		330	100.30		291	88.45		332	100.91
C. Rio Claro	100	54.35		168	83.17		195	96.53		195	96.53
Carvalhópolis	23	46.00		43	107.50		43	107.50		42	105.00
Conc. Aparecida	74	57.81		145	131.82		144	130.91		103	93.64
Divisa Nova	60	71.43		84	123.53		82	120.59		77	113.24
Fama	5	20.00		20	86.96		24	104.35		31	134.78
Guaranésia	95	45.89		236	95.16		244	98.39		243	97.98
Guaxupé	387	61.23		645	105.56		708	115.88		633	103.60
Juruaia	73	89.02		90	84.11		128	119.63		131	122.43
Machado	285	58.52		519	102.57		463	91.50		461	91.11
Monte Belo	68	62.39		116	84.67		150	109.49		146	106.57
Muzambinho	120	50.21		264	112.34		243	103.40		253	107.66
Nova Resende	107	56.02		234	121.88		206	107.29		208	108.33
Paraguaçu	107	47.35		254	102.01		271	108.84		245	98.39
PoçoFundo	103	65.19		157	95.15		176	106.67		182	110.30
S. P. da União	14	33.33		52	130.00		59	147.50		57	142.50
Serrania	30	30.30		104	101.96		107	104.90		93	91.18
**Total**	**2878**	**52.72**		**5582**	**102.99**		**5576**	**102.88**		**5588**	**103.10**

**Figure 2:**
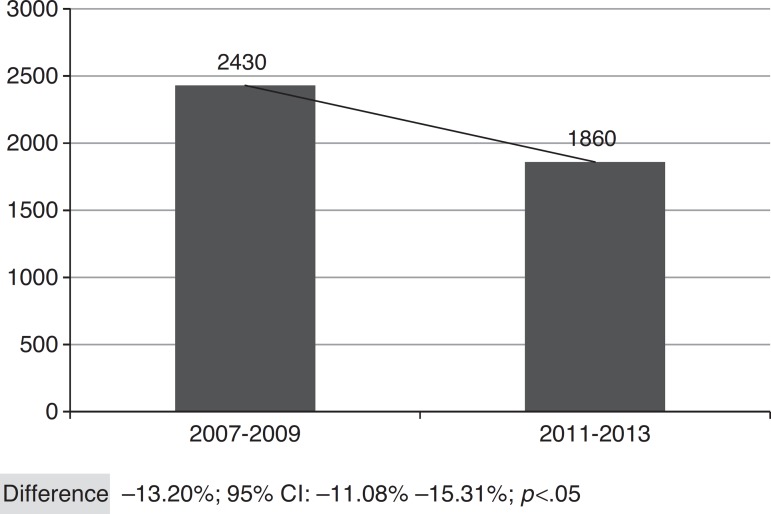
Total distribution of CAP cases in children, registered in the municipalities of SRS Alfenas, MG, Brazil before (2007-2009) and after (2011-2013) the start of immunization with 10-valent pneumococcal conjugate vaccine.

Fig. 2 shows the overall number of CAP admissions in periods pre- and post-introduction of the vaccine in PNI, considering all the municipalities of SRS/Alfenas. When considering the group of municipalities that comprises the SRS/Alfenas, there was a significant difference of−13.20% (95%CI:−44.08% to−15.31%; *p*<0.05) in the incidence of pediatric CAP hospitalizations between the study periods.

Multivariate analysis between vaccination status and the occurrence of CAP hospitalization in each of the 26 municipalities, adjusted for confounding variables, as well as of SRS as a whole, can be seen in Table 3.

**Table 3:** Analysis of the association between vaccination and the incidence of community-acquired pneumonia in children from 26 municipalities of the SRS/Alfenas, MG, Brazil, comparing the years 2007-2009 (before pneumococcal immunization) with the years 2011-2013 (after introduction of pneumococcal vaccine).

Municipality	PR	95%CI	*p*-value
Alfenas	1.07	0.81-1.42	>0.05
Alterosa	0.36	0.13-0.95	<0.05^a^
Arceburgo	0.22	0.05-0.90	<0.05^a^
Areado	0.59	0.24-1.47	>0.05
B.do Sul	0.33	0.07-1.65	>0.05
Botelhos	1.02	0.44-2.38	>0.05
Cabo Verde	1.26	0.79-1.99	>0.05
Campestre	0.71	0.35-1.41	>0.05
Campo do Meio	0.48	0.30-0.77	<0.05^a^
Campos Gerais	0.59	0.41-0.87	<0.05^a^
Carmo do Rio Claro	0.86	0.54-1.37	>0.05
Carvalhópolis	0.90	0.30-2.70	>0.05
Conceição da Aparecida	0.53	0.21-1.35	>0.05
Divisa Nova	1.88	0.32-10.90	>0.05
Fama	0.41	0.04-4.20	>0.05
Guaranésia	0.23	0.15-0.36	<0.05^a^
Guaxupé	1.27	1.08-1.50	<0.05^a^
Juruaia	0.57	0.37-0.88	<0.05^a^
Machado	0.63	0.40-0.90	<0.05^a^
Monte Belo	0.48	0.24-0.97	<0.05^a^
Muzambinho	0.88	0.41-1.91	>0.05
Nova Resende	0.72	0.44-1.19	>0.05
Paraguaçu	1.04	0.54-1.89	>0.05
Poço Fundo	0.37	0.15-0.89	<0.05^a^
São Pedro	3.15	0.67-14.70	>0.05
Serrania	1.14	0.44-2.90	>0.05
SRS Alfenas (Global)	**0.81**	**0.74-0.89**	**<0.05** ^a^

PR, prevalence ratio; 95%CI, 95% confidence interval.

a Significant.

**Figure 3:**
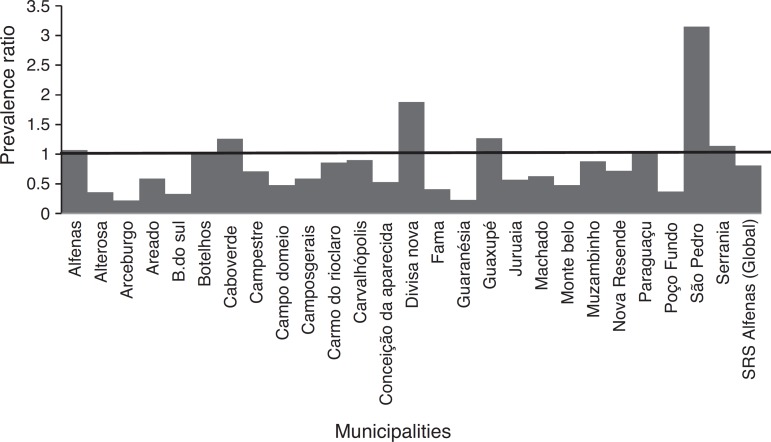
Representation of the prevalence ratio (PR) specific values relating to CAP hospitalization in children up to one year old, in each of the 26 municipalities, and considering the total value of SRS-Alfenas, MG, Brazil, after the introduction of the PCV-10 vaccine. Columns represent the values of prevalence ratio of CAP hospitalization after the introduction of the PCV-10 vaccine. Horizontal line represents the prevalence ratio=1; i.e., the non-changing line in the prevalence of CAP after introduction of the PCV-10 vaccine.

In the municipalities of Alfenas, Areado, Bandeira do Sul, Botelhos, Cabo Verde, Campestre, Carmo do Rio Claro, Carvalhópolis, Conceição da Aparecida, Divisa Nova, Fama, Muzambinho, Nova Resende, Paraguaçu, São Pedro da União, and Serrania there were no differences in CAP prevalence between vaccinated and unvaccinated children. In the municipalities of Alterosa, Arceburgo, Campo do Meio, Campos Gerais, Guaranésia, Juruaia, Machado, Monte Belo, and Poço Fundo there was a decrease in CAP hospitalization in vaccinated versus unvaccinated children, as shown in Fig. 3. In the municipality of Guaxupé there was a significant 27% increase in CAP hospitalization between the two study periods (pre- and post-vaccination). This phenomenon requires further clarification. In all the 26 municipalities, vaccination may have been responsible for a 19% reduction in hospitalizations due to CAP cases in children under one year old. During this period, there was no mortality from CAP in children under one year old in all the municipalities of the SRS/Alfenas.

## Discussion

This study showed a 19% reduction in CAP in children younger than one year between 2007-2009 and 2011-2013, corresponding to the periods before and after the introduction of the immunization schedule of PCV-10 in 2010 in the PNI, in 26 municipalities under the jurisdiction of SRS/Alfenas.

The Ministry of Health recommends 95% as the ideal rate of vaccination coverage able to give efficiency to the 10-valent pneumococcal vaccine to reduce cases of CAP and other invasive pneumococcal diseases.^11^^,^ 12 ^,^ 14 In this study, the average coverage rate in the year that the vaccine was introduced was only 52.7%; however, in 2011, 2012, and 2013 the rates were 104.0%, 102.9%, and 103.1%, respectively, which is above the recommended. It is expected, therefore, a reduction in the number of CAP cases and its consequences, such as hospitalization and mortality, which was actually seen. This finding is similar to that of a study by Afonso et al.^15^ reporting a reduction of pneumonia cases in cities where the coverage was over 95%, unlike the cities with lower coverage, such as São Paulo (75%) and Porto Alegre (85%), where such a reduction was not seen. In our study, as shown in Table 2, many municipalities showed vaccine coverage above 100%. This probably happened due to two important facts. The first refers to the intense seasonal migration in the municipalities during the coffee harvest period. Rural workers and their families who seek vaccination centers are vaccinated according to their respective age groups. The second fact could be the extensive rural area that many municipalities have. Thus, for many families, the distance from another city vaccination center is shorter than that of their own cities.

In addition to the aspects directly related to CAP morbidity and mortality, with impact on quality of life of children and their parents, there is the economic issue associated with the disease; for example, the cost linked to its management (spending on antibiotics, hospitalization, clinical tests, parental absence from work, and others). Thus, some studies have shown that children vaccination against pneumococcus is cost-effective from the perspective of society, reduces the total burden of pneumococcal disease, including CAP. Martí et al.^11^ analyzed the cost-effectiveness based on quality-adjusted life year (QALY) of PCV-10 in six Latin American countries, including Brazil, and reported that the incorporation of immunization is a cost-effective strategy in improving health standards of the pediatric population.

In analyzing the periods before and after the introduction of PCV-10 in PNI, as shown in Fig. 1, the number of CAP hospitalizations in the 26 selected municipalities had shown a decline from 2009 (pre-vaccine period), a fact that can certainly be explained by better sanitary conditions, increased household income, as well as gains in children health care seen in recent years throughout Brazil. Thus, vaccination against pneumococcus brought an increase to the decline observed in its post-introduction period since 2010.^16^^-^ 19

One limitation of the present study is the fact that it is an ecological study in which the observed unit is not the individual, but a group of people representing a portion of the population that is under the jurisdiction of SRS/Alfenas (children under one year old); however, we used official sources that have quality control—Datasus, SI-PNI, and Tabwin—available in the Regulation Center of SRS/Alfenas, but still it may have flaws, which was observed in the loss of records in the least 8.3% of cases in the post-vaccination period. Other limiting aspects of this study are related to potential confounding factors, such as the improvement in children's dietary patterns, observed in the last decade, contributing to a decreased incidence of severe CAP, in addition to vaccination, presence of some comorbidities, and differences in the prevalence of PAC between male and female.^18^^,^ 20 Finally, another limitation is the fact only children were assessed before the recommended booster shot between 12 and 15 months.

In the PCV-10 post-advent period, there was a decreased number of PAC in children under 12 months old in the region of SRS/Alfenas, MG, Brazil. In countries where pneumococcal conjugate and even polysaccharide vaccines were established and maintained with high vaccination coverage, the reduction of invasive pneumococcal disease is significant; the same result was found in this study, which showed a reduction in the number of CAP children hospitalized in the study period.
